# Vitamin D Supplementation is Associated with Increased Glutathione Peroxidase-1 Levels in Arab Adults with Prediabetes

**DOI:** 10.3390/antiox9020118

**Published:** 2020-01-29

**Authors:** Mohammed Ghouse Ahmed Ansari, Shaun Sabico, Mario Clerici, Malak Nawaz Khan Khattak, Kaiser Wani, Sara Al-Musharaf, Osama Emam Amer, Majed S. Alokail, Nasser M. Al-Daghri

**Affiliations:** 1 Chair for Biomarkers of Chronic Diseases, Riyadh Biochemistry Department, College of Science, King Saud University, Riyadh 11451, Saudi Arabia; mansari@ksu.edu.sa (M.G.A.A.); ssabico@ksu.edu.sa (S.S.); mkhattak@ksu.edu.sa (M.N.K.K.); kwani@ksu.edu.sa (K.W.); oamer1@ksu.edu.sa (O.E.A.); malokail@ksu.edu.sa (M.S.A.); 2 Department of Physiopathology and Transplantation, University of Milan, via Francesco Sforza, 35, 2022 Milan, Italy; mario.clerici@unimi.it; 3 IRCCS Fondazione Don Carlo Gnocchi, 20148 Milan, Italy; 4 Department of Community Health, College of Applied Medical Sciences, King Saud University, Riyadh 11451, Saudi Arabia; salmosharruf@ksu.edu.sa

**Keywords:** oxidative stress, glutathione peroxidase, vitamin D supplementation

## Abstract

Vitamin D supplementation may be used to lower oxidative stress. This interventional study aimed to investigate the effects of vitamin D supplementation on glutathione peroxidase 1 (GPx1) levels and other parameters in Arab adults with prediabetes. A total of 203 Saudi adults with prediabetes and vitamin D deficiency [intervention group, *N* = 146 (53 males and 93 females); control group, *N* = 57 (25 males and 32 females)] were included in this non-randomized, six-month intervention study. The intervention group received 50,000 international units (IU) cholecalciferol tablets once a week for two months, then twice a month for the next two months, followed by 1000 IU daily for the last two months. The control group received no supplementation. Serum 25(OH)D, lipid profile, glucose, C-reactive protein (CRP) and GPx1 were measured at baseline and after six months. Post-intervention, GPx1 concentrations increased significantly in the intervention group [17.3 (11.5–59.0) vs 26.7 (11.4–59.9) *p* < 0.01] while no changes were observed in the control group (*p* = 0.15). This significant increase in 25(OH)D and GPx1 levels persisted after adjusting for age and BMI. Stratification according to sex revealed that this favourable increase in GPx1 was true only for males (*p* = 0.002). In all groups, baseline GPx1 was inversely correlated with low density lipoprotein (LDL)-cholesterol (*r* = −0.26, *p* < 0.01) and body mass index (BMI) (*r* = −0.20, *p* < 0.05), while positively correlated with age (*r* = 0.18, *p* < 0.05) and systolic blood pressure (*r* = 0.19, *p* < 0.05). In conclusion, vitamin D supplementation favourably enhanced GPx1 levels in adult Arabs with prediabetes, particularly in males.

## 1. Introduction

Diabetes mellitus (DM)—a metabolic disorder characterized by insulin resistance and pancreatic β-cell damage—is one of the most common diseases in modern times, affecting around 451 million people globally and is expected to reach 693 million by 2045 [1]. In parallel, the pandemic of vitamin D deficiency has become a global health concern affecting people of all ages [2]. Saudi Arabia alone has an estimated population of 7 million living with DM in addition to 3 million with prediabetes, with an overall prevalence of vitamin D deficiency (<50 nmol/L) at 81.0% (confidence interval 95% 68.0–90.0) from 2011 to 2016 [3,4]. Sedentary lifestyle, environmental factors and genetic predisposition are considered major factors in the development of DM in the region [5,6,7,8,9,10]. 

Chronic hyperglycemia in DM induces excess production of free radicals [11]. Elevated oxidative stress caused by reactive oxygen species (ROS) plays a causal role in the pathogenesis of DM and related complications [12,13] via increased insulin resistance or impaired insulin secretion [14,15]. Oxidative stress can be defined as the imbalance between production and degradation of ROS [16]. Several studies have shown that it acts as a triggering factor for an array of human disorders including DM [13], cardiovascular disorders [17,18], cancers [19,20], neurodegenerative disorders [21] and autoimmune diseases [22]. 

Traditionally, 25-hydroxy vitamin D (25(OH)D) was considered essential in calcium homeostasis for optimal skeletal health, but recent scientific advances revealed its extraskeletal functions and associations with various clinical conditions [23,24,25]. Vitamin D deficiency has been considered a risk factor in type 1 diabetes mellitus (T1DM) and type 2 diabetes mellitus (T2DM) [26,27,28]. On the other hand, glutathione peroxidase (GPx) family plays a crucial role in the enzymatic antioxidant defense system. GPx1 is a ubiquitously expressed abundant isoform of the GPx family and acts as a protagonist in detoxifying harmful lipid-peroxides by converting hydrogen peroxide to lipid alcohol and water respectively, using glutathione as an electron donor [29,30].

Several lines of evidence highlight the protective role of GPx1 in patients with DM [31,32,33]. Similarly, some studies have suggested that vitamin D may have antioxidant properties [34]. Evidence from our previous study suggested that vitamin D supplementation maybe a promising adjuvant therapy for T2DM patients [35]. However, we found a paucity of vitamin D interventional studies with regards to antioxidant activities. We hypothesize that improving vitamin D status may favourably regulate GPx1 activity and may, therefore, delay the progression of individuals with prediabetes to T2DM. Hence the present study aimed to determine the effects of vitamin D supplementation on GPx1 levels in Saudi adults with prediabetes.

## 2. Materials and Methods

### 2.1. Participants

In this 6-month interventional study, a total of 257 adult Saudis aged 30–60 years, with vitamin D deficiency [25(OH)D < 50 nmol/L], were randomly selected from the Vitamin D Database of the Chair for Biomarkers of Chronic Diseases (CBCD), King Saud University (KSU), Riyadh, Kingdom of Saudi Arabia (KSA) and were assigned in the intervention group. A separate group of 57 adult Saudis (age- and BMI-matched) with vitamin D deficiency were also selected from the database. Briefly, this database was based on a capital-wide, multi-centre observational study done in primary health care centres (PHCCs) in Riyadh, Saudi Arabia, [36,37]. Written informed consents were obtained prior to inclusion in the study. Ethical approval was obtained from the Institutional Review Board (IRB) of King Saud Medical City (E-15-1667), Riyadh, Saudi Arabia. Participants answered a questionnaire that included demographic information, present and past medical history.

#### Exclusion Criteria

**Figure 1:**
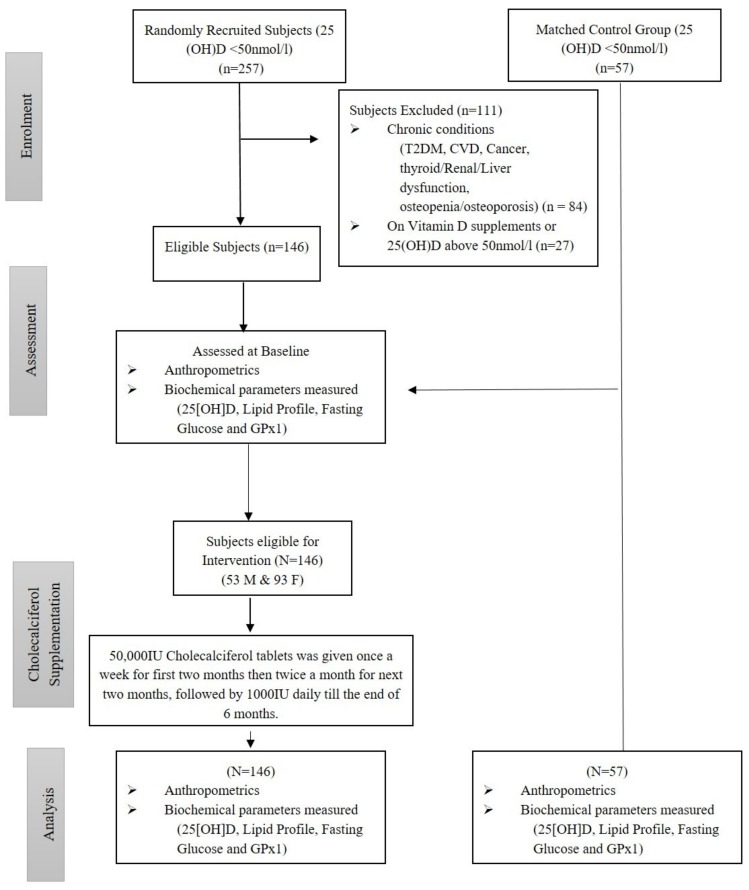
Modified CONSORT diagram for non-randomized design.

Participants with chronic clinical conditions (T2DM, cardiovascular diseases (CVD), cancer, gastrointestinal disease, osteopenia/osteoporosis, thyroid, liver and renal dysfunction), on any medication or vitamin D supplementation and whose baseline 25(OH)D levels were above 50 nmol/L were excluded from the study. A total of 84 participants were excluded because of the presence of the mentioned conditions for exclusion in their data file and another 27 participants were excluded for having baseline 25(OH)D levels above 50 nmol/L. All excluded participants(*N* = 111) were from the intervention group. Hence, 230 participants [intervention group, *N* = 146 (53 males and 93 females); control group, *N* = 57 (25 males and 32 females)] qualified for this interventional study (Figure 1).

### 2.2. Anthropometry and Biochemical Assessments

Anthropometric variables (waist circumference, hip circumference, waist to hip ratio (WHR), body mass index (BMI), systolic and diastolic blood pressure) and biochemical parameter (25[OH] D, fasting glucose and lipid profile) measurements were done at baseline and post-intervention by following the procedure and protocols, as described in our previous study [38].

Serum levels of GPx1 and CRP were measured using ELISA kits (Abcam^®®^, Cambridge, UK and R&D SYSTEMS^®®^, Minneapolis, MN, USA, respectively) following manufacturers’ instructions. The variations in intra-assay and inter-assay as obtained were 4.17% and 9.8% for GPx1 and 5.5–6.5% for CRP respectively. To minimize inter-assay variability, all samples were analyzed simultaneously and the actual variations were well within the inter- and intra-assay ranges.

### 2.3. Intervention

The intervention group received oral 50,000IU cholecalciferol tablets (VitaD50000) (Synergy pharma, Dubai, UAE) given once a week for the first two months then twice a month for the next two months, followed by 1000 IU (VitaD1000) (Synergy pharma, Dubai, UAE) daily for the last two months. Participants were regularly encouraged through Short Message Service (SMS) to take the recommended vitamin D dose. To ensure compliance, participants were requested to return unused tablets every follow-up visit (if any) before a refill of supplements was given. Dosages were based on the national recommendations for the management of vitamin D deficiency [39]. The control group did not receive any vitamin D supplementation but were given advice on how to increase vitamin D status non-pharmacologically (increased sunlight exposure and dietary intake of vitamin D-rich foods).

### 2.4. Statistical Analysis

Data were analyzed using SPSS version 21.0, IBM (Armonk, NY, USA). Mean ± standard deviation (SD) were used to present normal variables, while the median (1st and 3rd) percentiles were used for non-normal variables. All categorical variables were presented in percentages (%). Kolmogorov–Smirnov test was applied to check the normality. Non-normal variables were transformed prior to parametric testing. Paired T-Test and Wilcoxon tests were used to check the mean and median differences (changes) for Gaussian variables and non-Gaussian variables, respectively. Repeated measures ANOVA, was used to determine group and interaction effects. Pearson’s correlation analysis was done to determine the correlation between GPx1 and select variables. A *p*-value <0.05 was considered statistically significant. 

## 3. Results

A total of 203 (78 males and 125 females) vitamin D deficient Saudi adults participated in this six-month interventional study. Baseline comparisons of groups are presented in supplementary Table S1. There were no significant differences in all the parameters assessed in both groups at baseline. Table 1 shows the clinical characteristics of groups at pre- and post-intervention. Within-group comparisons in the intervention group showed a significant increase in glucose (*p* = 0.04), 25(OH)D (*p* < 0.01) and GPx1 (*p* < 0.01) overtime. In the control group, there was a significant increase in total cholesterol (*p* = 0.03) and HDL-cholesterol (*p* < 0.001) with a subsequent decrease in 25(OH)D (*p* = 0.01). Between-group comparisons revealed a clinically significant improvement in 25(OH)D (*p* < 0.001) and GPx1 (*p* < 0.01) in favour of the intervention group. The rest of the between-group comparisons were not significant (Table 1).

Table 2. Shows the within- and between-group comparisons of both groups according to sex. As expected, serum 25(OH)D significantly increased over time and between-groups in favour of the intervention group in both sexes. This clinically significant increase persisted even after adjusting for age and BMI (*p* < 0.001). With regard to GPx1 however, the significant increase over time was observed in male subjects of the intervention group and this effect remained significant in between-group comparisons even after adjusting for age and BMI (*p* = 0.023).

**Figure 2:**
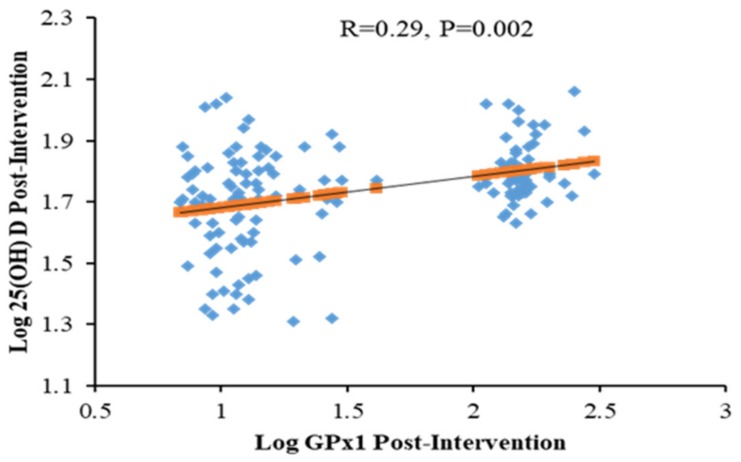
Relationship between Log 25(OH) D and GPx1 in all participants (Post-intervention).

Table 3 shows the bivariate correlation coefficients of GPx1 and other parameters for all participants at different time points according to groups. Overall and at baseline, GPx1 was inversely correlated with LDL-cholesterol (*r* = −0.26, *p* < 0.01) and BMI (*r* = −0.20, *p* < 0.05), while positively correlated with age (*r* = 0.18, *p* < 0.05) and systolic BP (*r* = 0.19, *p* < 0.05). In the intervention group, baseline GPx1 was inversely correlated with LDL-cholesterol (*r* = −0.22, *p* < 0.05) and total cholesterol (*r* = −0.19, *p* < 0.05) as well as positively correlated with WHR (*r* = 0.25, *p* < 0.01) systolic BP (*r* = 0.21, *p* < 0.05) and diastolic BP (*r* = 0.26, *p* < 0.01). In the control group, baseline GPx1 levels were positively correlated with triglycerides (*r* = 0.33, *p* < 0.05). Lastly, Figure 2 shows the significant positive association between post-intervention levels of 25(OH)D and GPx1 in the intervention group (*R* = 0.29; *p* = 0.002). This post-intervention association was not observed in controls.

## 4. Discussion

This interventional study revealed a clinically significant improvement in the vitamin D status and GPx1 levels after six months of vitamin D supplementation in favour of the intervention group. HDL-cholesterol increased significantly among female participantss of the control group while fasting blood glucose modestly increased in male participantss of the intervention group. These changes were not clinically significant in between-group comparisons. In all participants, GPx1 levels were significantly and inversely correlated with LDL-cholesterol in both time points and positively with 25(OH)D only at post-intervention. Finally, the significant improvement in GPx1 levels post-vitamin D supplementation were observed only in males.

T2DM is characterized by insulin resistance and/or insulin secretory dysfunction which can be influenced by vitamin D status. In the present study, serum 25(OH)D levels significantly improved in the intervention group post-vitamin D supplementation and these beneficial changes may help in improving glycemic control [35,40,41]. Other studies however revealed neither improvement in glycemic status [42] nor decreased diabetes risk in response to vitamin D supplementation [43].

At baseline, low GPx1 levels were observed in all participants. Similar results were reported in T2DM patients [44,45] and in the Athero Gene study of CVD patients, suggesting that patients with low erythrocyte GPx1 activities had an increased incidence of recurrent events [46,47]. Elevated ROS act as second messengers which signal inflammasome activation [48]. This may be reflected in our results with females, where expression of the inflammatory protein CRP was modestly reduced post-intervention.

In the present study, the significant positive associations of GPx1 to known cardiometabolic factors such as age, BMI and blood pressure, as well as the inverse association with LDL-cholesterol, highlight the known protective role of GPx1 in decreasing the chronic sub-inflammatory status observed in diseases such as T2DM [31,32,33]. Our findings showed a significant positive correlation between GPx1 levels with 25(OH)D levels, and may, therefore, reduce oxidative stress in patients with prediabetes. These findings are in line with a recent study on decreased oxidative stress in paraspinal skeletal muscles of low back pain patients who received vitamin D supplementation [49]. Interestingly, only LDL-cholesterol was associated with GPx1 in the present study. A similar study, however, done among overweight and obese populations of central Mexico revealed significant associations between HDL and triglycerides with GPx [50]. These differences may indicate that GPx is highly variable in human populations having different health conditions as well as ethnicity [51].

Lastly, the favorable increase in GPx1 observed only in males post-vitamin D intervention highlight the sex-specific extra-skeletal effects of vitamin D correction. Our previous observation involving more than 3000 Saudi adolescents and adults revealed that vitamin D deficiency and its association with cardiometabolic risk factors were mostly limited to males. This led us to believe that vitamin D correction may prove more beneficial to men than women, at least in terms of extraskeletal benefits [52]. One explanation that we have also recently documented at the proteomic level is that the conversion of 25(OH)D to its active form, 1,25(OH)D_2_ is higher in men than women, and this can be linked to sex hormone metabolism [53].

The authors acknowledge certain limitations. Even though baseline characteristics between control and intervention group were not significant, randomization was not done in the present study and as such, there is a chance of bias in reported changes, post-intervention. Other antioxidant markers were unfortunately not included in the present study and this could have enhanced, if not supplemented our hypothesis that vitamin D has antioxidant properties. Nevertheless, this study is the first of its kind to explain the effects of vitamin D supplementation on GPx1 levels.

## 5. Conclusions

Vitamin D supplementation modulates GPx1 levels that can favourably benefit vitamin D deficient patients with prediabetes, particularly males. The present study should be further explored to see how vitamin D supplementation stimulates the antioxidant system by investigating other antioxidant markers, including the trace minerals.

## Figures and Tables

**Table 1:** Clinical Characteristics of Groups Before and After 6 Months of Intervention.

Parameters	Intervention				Control				*p*-Value
	Before	After	Change	*p*-Value	Before	After	Change	*p*-Value	
**N (M/F)**	146 (53/93)				57 (25/32)				
Total Cholestrol (mmol/L)	5.1 ± 1.2	5.12 ± 1.2	−0.02 (−0.2–0.16)	0.52	5.07 ± 1.0	5.51 ± 1.2	0.44 (0.13–0.75)	0.03	0.19
HDL-Cholestrol (mmol/L)	1.0 ± 0.4	1.15 ± 0.40	0.11 (0.02–0.19)	0.14	1.05 ± 0.5	1.30 ± 0.4	0.22 (0.08–0.37)	<0.001	0.71
LDL-Cholestrol (mmol/L)	3.2 ± 0.9	3.14 ± 0.9	−0.11 (−0.3–0.06)	0.12	3.30 ± 0.8	3.44 ± 0.9	0.14 (−0.14–0.41)	0.65	0.38
Triglycerides (mmol/L) #	1.5 (1.0–2.1)	1.49 (1.1–1.9)	0.01 (−0.4–0.4)	0.56	1.18 (0.8–1.8)	1.51 (0.9–2.3)	0.24 (−0.26–0.9)	0.12	0.29
Glucose (mmol/L)	5.5 ± 0.9	5.6 ± 0.9	0.07 (−0.1–0.23)	0.04	5.35 ± 0.10	5.48 ± 0.8	0.13 (−0.17–0.44)	0.98	0.55
CRP (µg/mL)#	20.6 (4.5–49.5)	39.9 (14.9–75.9)	0.27 (−10.5–4.5)	0.39	17.7 (5.2–40)	34.7 (8.0–80.4)	0.13 (−20.1–17.7)	0.22	0.24
25(OH)D (nmol/L)	32.5 ± 11.6	66.2 ± 18.01	33.7 (30.6–36.8)	<0.01	31.9 ± 15.3	29.10 ± 12.4	−2.8 (−6.2–0.8)	0.01	<0.001
GPx1 (ng/mL)	17.3 (11.5–59.0)	26.7 (11.4–59.9)	7.5 (−1.7–8.2)	<0.01	14.6 (7.6–56)	16.3 (8.5–66.3)	1.9 (−4.5–6)	0.15	0.01

**Table 2:** 25(OH) D and GPX-1 at Baseline and After 6 Months of Intervention.

Parameter	Males		Females		Group Effect	Group Effect (Adjusted for age & BMI)
	Intervention (*N* = 53)	Control (*N* = 25)	Intervention (*N* = 93)	Control (*N* = 32)		
25 (OH) D (nmol/Ll)						
**Baseline**	34.9 ± 10.8	38.6 ± 13.7	30.9 ± 14.5	29.2 ± 16.2	<0.001	<0.001
6 months	60.1 ± 20.2	32.8 ± 9.5	54.3 ± 24.5	27.4 ± 12.9		
Change (1st–3rd) percentile	26.0 (19.8–32.2)	−2.5 (-22.4–7.3)	21.1 (15.1–26.1)	−1.2 (−10.9–7.9)		
Time effect	<0.001		<0.001			
Time effect (Adjusted)	<0.001		<0.001			
GPx1 (ng/mL)					< 0.001	<0.023
Baseline	24.6 (14–140)	16.6 (13.4–23.5)	14.9 (11.1–41.6)	11.1 (9.8–13.)		
6 months	30.1 (12–140)	18.6 (10.2–27.2)	16.1 (10.1–44.5)	11.5 (11.5–14.1)		
Change (1st–3rd) percentile	8.9 (2.9–14.9)	2.04 (−5.7–6.4)	6.9 (2.7–11.1)	0.16 (−1.6–2.8)		
Time effect	0.004		0.06			
Time effect (Adjusted)	0.002		0.57			

**Note:** Data presented as mean ± standard deviation, Median (1st and 75th) percentiles and mean and median change (95% CI); Adjusted for age and BMI; significant at *p* < 0.05.

**Table 3:** Baseline GPx1 Associations in All Groups.

Parameters	All	Intervention	Control
*N*	203	146	57
Age (year)	0.18 *	0.19	0.05
Body Mass Index (BMI) (kg/m^2^)	−0.20 *	−0.17	0.21
Waist Hip Ratio (WHR)	0.20	0.25 **	0.25
Systolic BP (mmHg)	0.19 *	0.21 *	0.20
Diastolic BP (mmHg)	0.16	0.26 **	0.14
Total Cholesterol (mmol/L)	−0.11	−0.19 *	−0.04
HDL-Cholesterol (mmol/L)	0.12	0.15	0.18
LDL-Cholesterol (mmol/L)	−0.26 **	−0.22 *	−0.20
Triglycerides (mmol/L) #	0.10	0.01	0.33 *
Glucose (mmol/L)	0.16	0.04	0.16
25(OH) D (nmol/L)	0.06	0.03	0.20
CRP (µg/mL) #	−0.10	−0.08	0.10

**Note:** Data presented as a coefficient (R); # denotes Non-Gaussian;* denotes significance at 0.05 level; ** denotes significance at 0.01 level.

## Supplementary Materials

The following are available online at https://www.mdpi.com/2076-3921/9/2/118/s1, Table S1: Baseline Characteristics of Intervention and Control Groups.
