# Supplementary material for: Vitamin D Supplementation is Associated with Increased Glutathione Peroxidase-1 Levels in Arab Adults with Prediabetes
Source: Antioxidants (Basel). 2020 Jan 29;9(2):118. doi: 10.3390/antiox9020118 (PMC7070325; doi:10.3390/antiox9020118)
Supplement: Supplementary file 1 [file antioxidants-09-00118-s001.pdf]

Supplementary Table 1. Baseline Characteristics of Intervention and Control Groups

**Parameters**	**Intervention**	**Control**	**P-value**
N (M/F)	146 (53/93)	57 (25/32)	0.18
Age (year)	38.9 ± 10.9	37.6 ± 10.4	0.90
BMI (kg/m^2)	29.6 ± 4.8	28.2 ± 4.7	0.30
WHR	0.92 ± 0.12	0.93 ± 0.13	0.86
Systolic BP (mmHg)	126.5 ± 13.4	122.7 ± 15.0	0.23
Diastolic BP (mmHg)	78.6 ± 8.7	76.4 ± 8.9	0.97
Total Cholesterol (mmol/l)	5.13 ± 1.2	5.07 ± 1.0	0.40
HDL-Cholesterol (mmol/l)	1.04 ± 0.41	1.05 ± 0.5	0.84
LDL-Cholesterol (mmol/l)	3.25 ± 0.9	3.30 ± 0.8	0.72
Triglycerides (mmol/l) #	1.46 (1.01-2.1)	1.18 (0.8-1.8)	0.43
Glucose (mmol/l)	5.5 ± 0.9	5.35 ± 0.10	0.09
CRP (µg/ml)#	20.6 (4.5-49.5)	17.7 (5.2-40)	0.50
25(OH)D (nmol/l)#	32.5 ± 11.6	31.9 ± 15.3	0.76
GPX (ng/ml)	17.3 (11.5-59.0)	14.6 (7.6-56)	0.18

Note: P-value significant at <0.05.
